# Accelerated QT adaptation following atropine‐induced heart rate increase in LQT1 patients versus healthy controls: A sign of disturbed hysteresis

**DOI:** 10.14814/phy2.15487

**Published:** 2022-11-02

**Authors:** Pia Dahlberg, Karl‐Jonas Axelsson, Steen M. Jensen, Gunilla Lundahl, Farzad Vahedi, Rosie Perkins, Lennart Gransberg, Lennart Bergfeldt

**Affiliations:** ^1^ Department of Molecular and Clinical Medicine Institute of Medicine, Sahlgrenska Academy, University of Gothenburg Gothenburg Sweden; ^2^ Region Västra Götaland, Department of Cardiology Sahlgrenska University Hospital Gothenburg Sweden; ^3^ Department of Public Health and Clinical Medicine, and Heart Centre Umeå University Umeå Sweden

**Keywords:** atropine, cardiac memory, hysteresis, long QT syndrome, QT adaptation

## Abstract

Hysteresis, a ubiquitous regulatory phenomenon, is a salient feature of the adaptation of ventricular repolarization duration to heart rate (HR) change. We therefore compared the QT interval adaptation to rapid HR increase in patients with the long QT syndrome type 1 (LQT1) versus healthy controls because LQT1 is caused by loss‐of‐function mutations affecting the repolarizing potassium channel current I_Ks_, presumably an important player in QT hysteresis. The study was performed in an outpatient hospital setting. HR was increased in LQT1 patients and controls by administering an intravenous bolus of atropine (0.04 mg/kg body weight) for 30 s. RR and QT intervals were recorded by continuous Frank vectorcardiography. Atropine induced transient expected side effects but no adverse arrhythmias. There was no difference in HR response (RR intervals) to atropine between the groups. Although atropine‐induced ΔQT was 48% greater in 18 LQT1 patients than in 28 controls (*p* < 0.001), QT adaptation was on average 25% faster in LQT1 patients (measured as the time constant τ for the mono‐exponential function and the time for 90% of ΔQT; *p* < 0.01); however, there was some overlap between the groups, possibly a beta‐blocker effect. The shorter QT adaptation time to atropine‐induced HR increase in LQT1 patients on the group level corroborates the importance of I_Ks_ in QT adaptation hysteresis in humans and shows that LQT1 patients have a disturbed ultra‐rapid cardiac memory. On the individual level, the QT adaptation time possibly reflects the effect‐size of the loss‐of‐function mutation, but its clinical implications need to be shown.

## Graphical abstract

Accelerated QT adaptation after atropine in LQT1. During continuous Frank vectorcardiography, an intravenous bolus injection of atropine (0.04 mg^*^kg‐1 body weight) was used to induce a rapid heart rate increase in LQT1 patients and healthy controls with similar proportions of women and men. Although the heart rate reaction was similar (graph to the left) and the change in QT was on average 48% larger (not shown) in LQT1 patients, the QT adaptation time was on average 25% shorter in LQT1 patients (graph to the right). The graphs are from individuals with results closest to the group average.

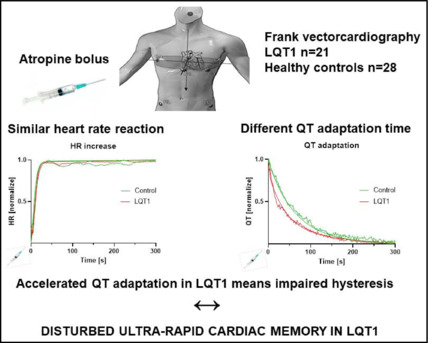

## 1 INTRODUCTION

Hysteresis is a ubiquitous regulatory phenomenon (Noori, 2014). In the present context of cardiac electrophysiology, the focus is on ventricular repolarization hysteresis reflected by the QT interval adaptation to heart rate (HR) increase. The electrophysiological benefit of QT hysteresis in repolarization adaptation is presumably to provide electrical stability through smooth changes in regional action potential duration (Berger, 2004; Eisner et al., 2009). Furthermore, it provides a smooth adaptation of the relation between the time for ventricular filling and emptying as well as the time for coronary perfusion (Berger, 2004). QT hysteresis, which is a salient feature of the ultra‐rapid cardiac memory (Rosen & Bergfeldt, 2015), is the result of adaptation in several ion channels for membranous in‐ and outward directed currents (I_Na_, I_CaL_, I_Kr_, I_Ks_) and ion pumps (i.e. Na/K‐ATPase) (Eisner et al., 2009; Pueyo et al., 2010). The physiology and patho‐physiology of QT hysteresis is therefore not only of theoretical but potentially also of clinical importance since the involved currents and ion pumps can be affected by disease processes as well as by pharmacological substances.

The hysteresis of QT adaptation in individual hearts has been explored in several studies by investigating the QT–RR relationship during exercise testing or from Holter recordings. Different measures of hysteresis were applied and provided conflicting results, as recently reviewed (Gravel et al., 2018). More consistent results on the physiology of QT hysteresis have been obtained by changing the HR either by incremental or sudden onset/offset of atrial or ventricular pacing, which, however, requires cardiac catheterization or the use of permanently implanted pacemakers (Axelsson et al., 2020; Axelsson, Gransberg, Lundahl, Vahedi, & Bergfeldt, 2021; Franz et al., 1988; Lau et al., 1988; Seethala et al., 2011). Little is, however, known about the QT hysteresis in disease states. We chose to study patients with the long QT syndrome (LQTS) which belongs to the group of diseases known as channelopathies. It has a prevalence of >1 per 2000 and is a major risk factor for sudden arrhythmic cardiac death, especially in the young (Amin et al., 2013; Priori et al., 2015; Schwartz et al., 2012). LQT1 is the most common subtype, accounting for ~50% of genotype‐confirmed LQTS, and is caused by loss‐of‐function mutations in *KCNQ1*, the gene encoding the ion channel protein responsible for the slow repolarizing potassium current I_Ks,_ one of the currents involved in QT hysteresis (Amin et al., 2013; Eisner et al., 2009; Pueyo et al., 2010). Because few LQT1 patients have pacemakers and catheterization for the present research purpose cannot be ethically justified, we chose a pharmacological method for rapidly increasing HR. Atropine was introduced for this purpose >50 years ago, and we used doses proven to be safe in healthy persons and in patients with proven or suspected sinus node disease below the age of 60 years (Bergfeldt et al., 1996; Jose & Taylor, 1969; Vahedi et al., 2012).

The purpose of this study was thus to test the hypothesis that QT hysteresis is affected in patients with LQT1 with loss‐of‐function mutations reducing I_Ks_. To optimize measurement precision, we used Frank vectorcardiography (VCG) (Frank, 1956). This methodology allows us to measure the QT interval from one spatial (aka global) QRST complex, which has been shown to be superior to standard 12‐lead ECG for diagnosing LQTS (Diamant et al., 2010). To induce a rapid increase in HR in a standardized way and to minimize technical noise due to body movements, we gave atropine in doses sufficient to abolish parasympathetic influence on the sinus node while continuously recording VCG (Jose & Taylor, 1969; Vahedi et al., 2012).

## 2 METHODS

### 2.1 Study subjects

LQTS patients were recruited from the cardiogenetic outpatient clinics at Sahlgrenska University Hospital and Umeå University Hospital, Sweden. LQTS patients with a pathogenic gene variant in KCNQ1, without proven disease‐related symptoms and without acute or chronic illness apart from LQTS were included in this study; one patient had losartan‐treated hypertension. As reference, we performed an identical analysis of recordings from healthy individuals who underwent the same intervention as the LQT1 patients in an earlier study (Vahedi et al., 2012).

### 2.2 Procedure and protocol

The study was performed in a hospital setting. Eight surface electrodes were applied for Frank VCG. A peripheral venous cannula was inserted. VCG was recorded continuously with the individual resting with closed eyes in a supine position throughout the test procedure. After at least 5 min of VCG recording during silence, an intravenous bolus injection of atropine (0.04 mg/kg; maximum 5 mg) was administered over 30 s. The VCG recording continued for at least 20 min. The patients stayed for observation in the clinic for another 2–3 h.

### 2.3 Electrocardiographic recordings and measurements

VCG was recorded with a CoroNet II system (Ortivus, Danderyd, Sweden). The signals were sampled at 500 Hz, with an amplifier bandwidth of 0.03–170 Hz. QT and QT_peak_ intervals were used to measure ventricular repolarization duration and were analyzed beat‐by‐beat together with each RR interval (instantaneous HR), using customized software. The system calculates a global QRST complex from the three QRST complexes in the orthogonal X, Y, and Z directions with automatically set annotation points for onset, offset, and peak of the QRS complex and T wave. The QT_peak_ interval was measured from QRS onset to T peak (maximum T amplitude) and the QT interval from QRS onset to T‐wave end defined by the tangent method (Lundahl et al., 2020; Vink et al., 2018). QTc was HR corrected according to Bazett (Dahlberg et al., 2021).

### 2.4 Measurement of repolarization adaptation

QT and QT_peak_ adaptation was evaluated in relation to the change in RR intervals (beat‐to‐beat HR), which was the input in this regulatory process. The starting point of the response to atropine was therefore identified as the start of the rapid change in RR, and the analyzed period was 5 min after the starting point.

**FIGURE 1:**
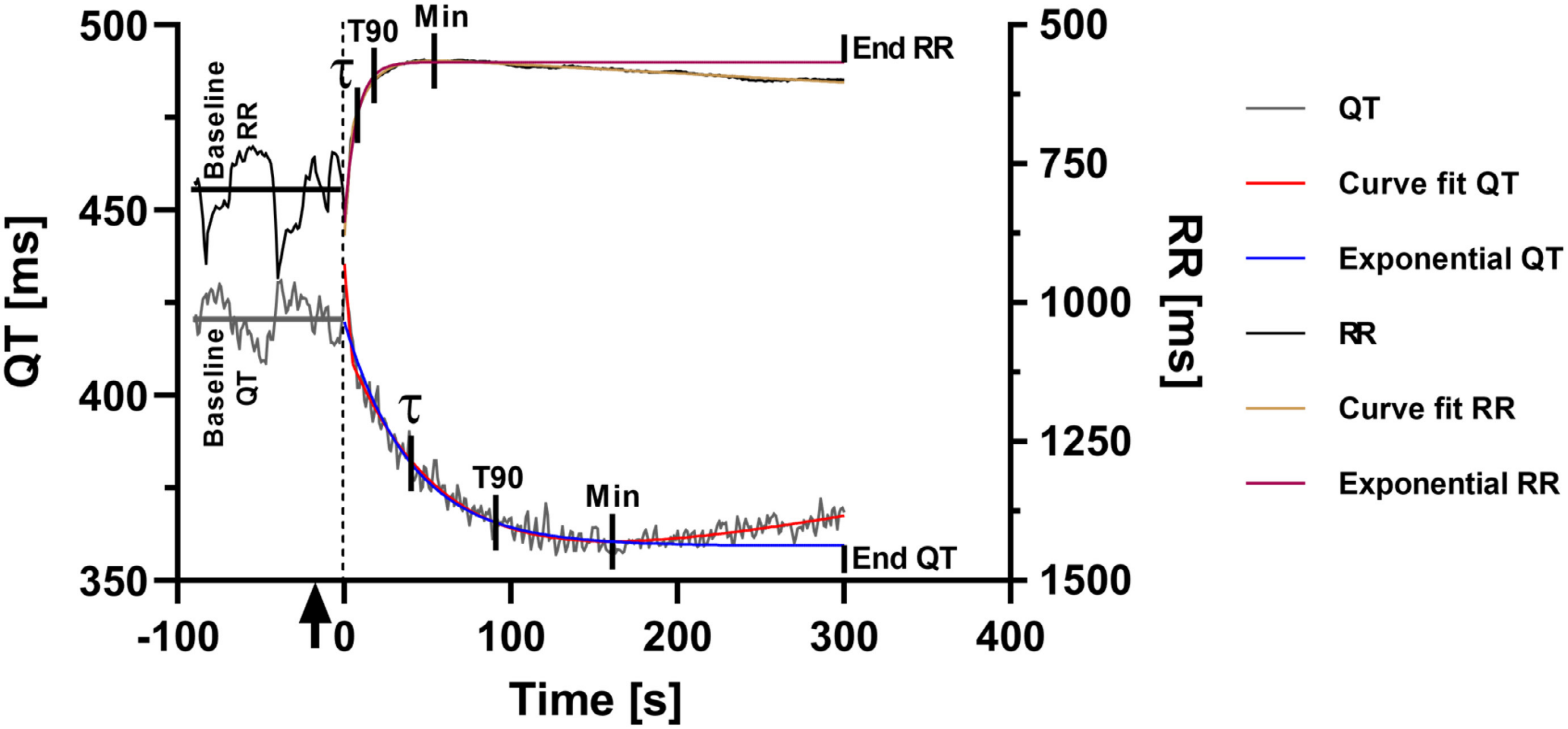
Beat‐to‐beat immediate heart rate (RR interval) and QT adaptation following an intravenous atropine bolus dose (arrow) in an LQT1 patient. The time point 0 denotes the start of the RR response to atropine. RR intervals are plotted on the right Y axis, which is inversed to separate the curves (hence RR Min appears to be a maximum value). Baselines are the average of RR and QT, respectively, for 90 s before 0. T90: T90 End, Curve fit QT and RR: double exponential curve fit to identify the Min value (maximum change from baseline) which defines the End value in T90 End. Exponential QT and RR: mono‐exponential curve fit to define τ and T90 End.

We used two time measures to describe the RR response to atropine and the QT and QT_peak_ adaptation to changes in RR interval: τ (tau) and T90 End. These measures have been used in previous publications studying QT adaptation to sudden onset HR increase induced by cardiac pacing in humans (Seethala et al., 2011; Axelsson et al., 2021). The calculation of τ follows the principle of time constants for exponential functions based on the natural e‐logarithm where in this context ${\mathrm{QT}}_{t}={\mathrm{QT}}_{\text{baseline}}-\mathrm{\Delta QT}*\left(1-e^{-t/\tau }\right)$ for the QT adaptation in response to changing HR (Figure 1). The first value in the exponential function is equal to ${\mathrm{QT}}_{\text{baseline}}$ and ΔQT and ΔQT_peak_ are the maximum changes of QT and QT_peak_. τ is associated with the steepness or speed of change of the exponential curve and represents the time point when the exponential function has reached 1‐e^−1^ (~63%) of ΔQT and ΔQT_peak_, respectively (Axelsson et al., 2021). T90 End is the time from the RR reaction start to 90% of the end value and follows the example of measuring the action potential duration at 90% repolarization (APD_90_) (Axelsson et al., 2021; Bergfeldt et al., 2017).

Each cardiac cycle provided one set of individual data‐points for RR, QT, and QT_peak_ and the series of these data‐points were fitted to exponential curves (in Microsoft Excel's Problem Solver) with the mean‐square fit method. After the atropine injection and rapid change of data, the minimum RR, QT, and QT_peak_ values were followed by a slight rebound, i.e. the HR became slightly lower and the QT and QT_peak_ intervals slightly longer (Figure 1). This led to difficulties in finding a steady‐state end value. We therefore chose a two‐step procedure. First, to identify the minimum value with high precision and reproducibility which was used to define the end value, a curve fit based on a double‐exponential function was used. Second, a mono‐exponential function using data from start to the thus defined end value was used to define τ and T90 End (Figure 1).

All curve‐fits were created in the same way for patients and controls. Before any measurements were performed and to avoid bias, each curve‐fit was scrutinized by four of the authors together and had to be unanimously judged as technically satisfactory (K‐JA, GL, LG, LB).

### 2.5 Statistics

Data are presented as mean (SD), but nonparametric tests were used for between group comparisons to obtain robust results (Mann–Whitney U test). IBM Statistical Package for the Social Sciences (SPSS, version 24) and GraphPad Prism (version 9) were used for statistical calculations and graphical presentations.

#### 2.5.1 Study approval

The study was conducted in accordance with the Declaration of Helsinki and approved by the regional ethics committee in Gothenburg #1021–15. Written informed consent was obtained from all subjects.

## 3 RESULTS

### 3.1 Study participants and safety of atropine

In this study, we enrolled 21 LQT1 patients and, as a reference, we also analyzed de novo VCG recordings from 31 healthy controls obtained in an earlier study (Vahedi et al., 2012). In both patients and controls, we increased HR by administering an intravenous bolus dose of atropine (0.04 mg/kg body weight).

For technical reasons, recordings from 18 LQT1 patients (of 21; 86%) and 28 controls (of 31; 90%) were suitable for quantitative assessment of the adaptation process of QT and QT_peak_. Furthermore, the recording in one of the 18 LQT1 patients was not suitable for analysis of QT, and in another of QT_peak_, which resulted in *n* = 17 for these two measures (as indicated in figures and tables). For controls, three registrations were not suitable for analysis of QT_peak_ (hence *n* = 25). Demographic and clinical characteristics are described in Table 1. LQT1 patients were older and had higher mean arterial pressure and longer QTc than controls. Six patients were on continuous beta‐blocker therapy. In response to atropine, most patients and controls experienced transient dry mouth, accommodation difficulties, and tiredness. There were no arrhythmic adverse effects.

**TABLE 1:** Clinical and demographic characteristics of the study subjects

Study subject characteristics	LQT1 (*n* = 18)	Controls (*n* = 28)	*p*‐value
Men/women (no)	10/8	16/12	—
Age (years)	40 (14)	26 (4)	<0.001
Heart rate (bpm)	61 (11)	67 (11)	0.150
QTcBazett (ms)	438 (33)	395 (22)	<0.001
Body weight (kg)	86 (24)	72 (12)	0.031
MAP (mmHg)	95 (11)	73 (9)	<0.001
Beta‐blocker therapy (no)	6	0	—

*Note:* Data are shown as mean (SD). Mann–Whitney U test was used to test differences.

Abbreviations: Bpm, beats per min; MAP, mean arterial pressure.

### 3.2 Heart rate adaptation

**FIGURE 2:**
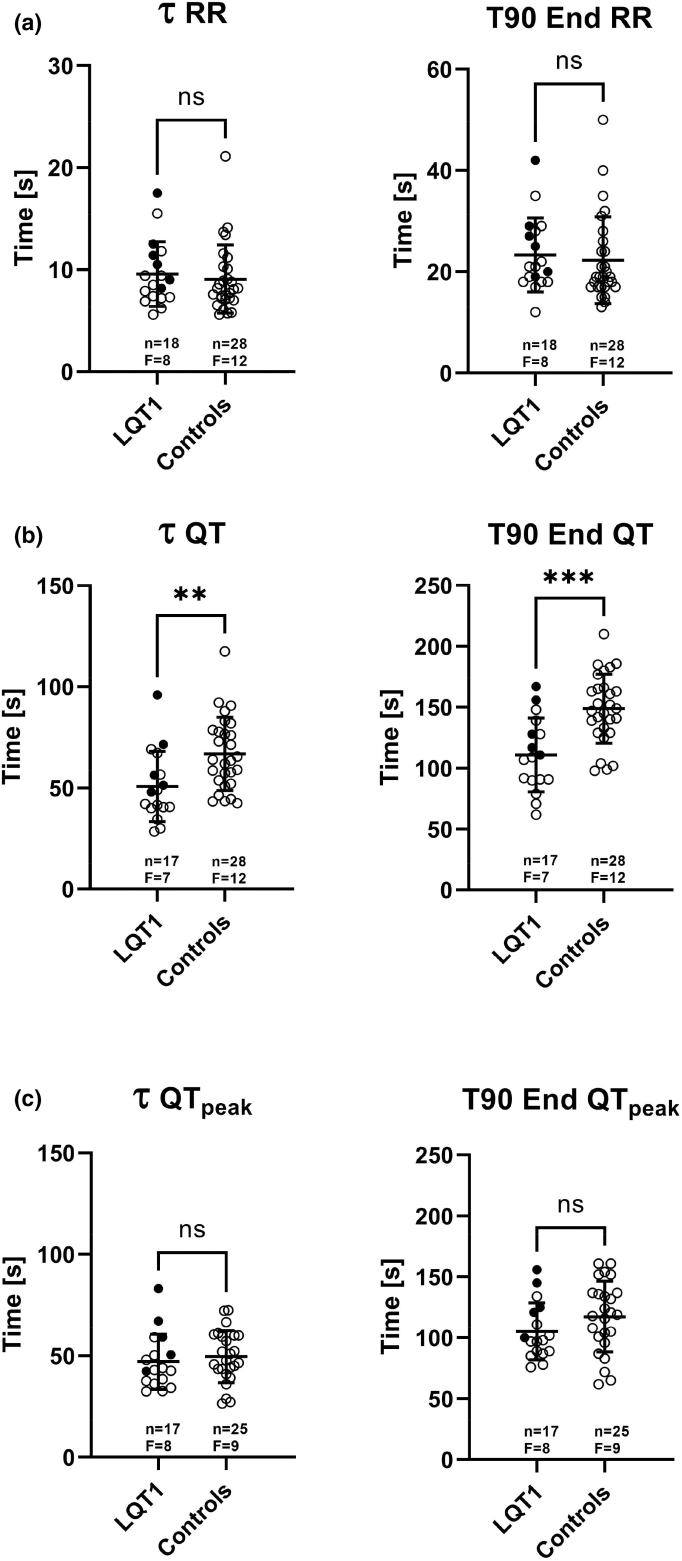
Comparison of adaptation time measures for RR (a), QT (b), and QT_peak_ (c) between LQT1 patients and healthy controls, following heart rate increase induced by a bolus injection of atropine. T90 End, the time to reach 90% of ΔRR, ΔQT or ΔQT_peak_; τ, the time constant of the exponential function fitted to the RR, QT, and QT_peak_ data. Filled circles denote LQT1 patients on beta‐blocker therapy. *p < 0.05, **p < 0.01, *** *p* < 0.001.

In response to atropine, a prompt rise in HR (T90 End on average 22–23 s) was observed in all individuals. Mean HR increased similarly in both groups (from 61 to 111 bpm in LQT1 patients and from 67 to 113 bpm in healthy controls). RR at baseline, RR end values after atropine, and time measures of the RR adaptation (τ and T90 End [Figure 1]) did not differ between the LQT1 patients and healthy controls (Figure 2, Table 2).

**TABLE 2:** Atropine‐induced changes in the instantaneous heart rate (RR interval) and the adaptation of ventricular repolarization duration measured as the QT and QT_peak_ intervals

Measures of heart rate and repolarization adaptation	LQT1		Controls		*p*‐value
	n		n		
RR baseline (ms)	18	1004 (172)	28	923 (140)	0.150
Heart rate baseline (bpm)		61 (11)		67 (11)	0.150
RR end value (ms)	18	545 (54)	28	533 (45)	0.458
Heart rate end value (bpm)		111 (11)		113 (10)	0.458
ΔRR (ms)	18	459 (143)	28	390 (118)	0.105
τ RR (s)	18	10 (3)	28	9 (3)	0.405
T90 End RR (s)	18	23 (7)	28	22 (9)	0.263
QT baseline (ms)	17	439 (50)	28	378 (28)	<0.001
QT end value (ms)	17	346 (35)	28	315 (17)	0.005
ΔQT (ms)	17	93 (23)	28	63 (19)	<0.001
τ QT (s)	17	51 (17)	28	67 (18)	0.002
T90 End QT (s)	17	111 (30)	28	149 (28)	<0.001
QT_peak_ baseline (ms)	17	356 (40)	25	303 (26)	<0.001
QT_peak_ end value (ms)	17	278 (30)	25	244 (14)	<0.001
ΔQT_peak_ (ms)	17	78 (16)	25	59 (17)	0.001
τ QT_peak_ (s)	17	47 (14)	25	50 (13)	0.254
T90 End QT_peak_ (s)	17	105 (23)	25	117 (29)	0.130

*Note:* Data are shown as mean (SD). Mann–Whitney U test was used to test differences. There were 18 LQT1 patients included but one missing for QT and another for QT_peak_; hence *n* = 17, and three controls missing for QT_peak_; hence *n* = 25.

Abbreviations: Bpm, beats per minute; T90 End, the time to 90% of the end value of the reaction; τ, the time constant for the mono‐exponential curve fit.

### 3.3 QT and QT_peak_ adaptation

The QT and QT_peak_ adaptation pattern to atropine‐induced HR increase showed a relatively short and rapid initial phase in most patients (*n* = 17) and controls (*n* = 23), but there was a considerable notch in the initial part of the curve in one patient and five controls (Figure S1). The subsequent rapid phase was, however, mono‐exponential regardless of the initial pattern; exclusion of those with the initial notch in the adaptation curve did not affect the results (Table S1).

**FIGURE 3:**
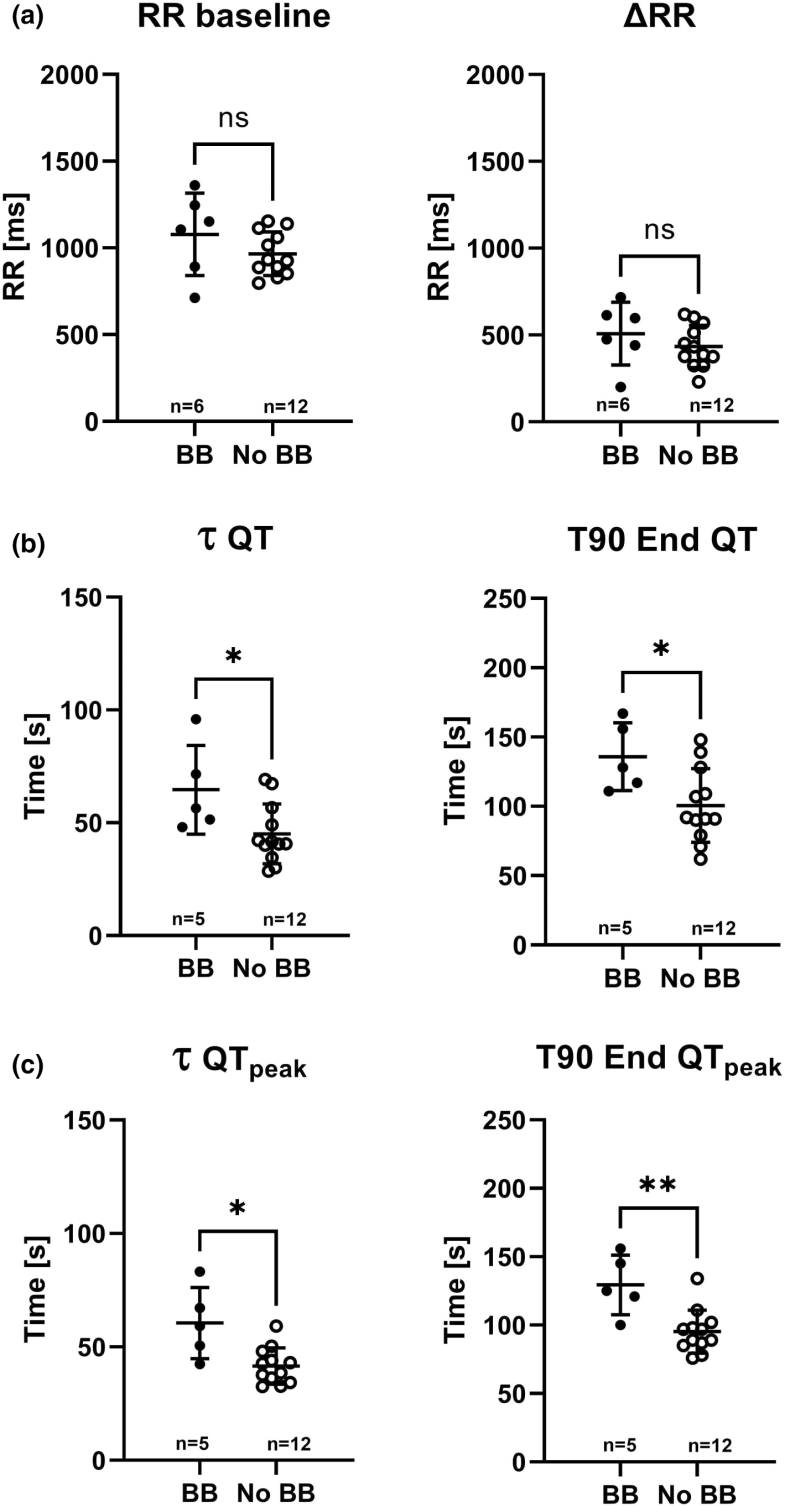
Comparison of RR (a), QT (b), and QT_peak_ (c) adaptation after atropine injection between LQTS patients type 1 with (filled circles) versus without (open circles) beta‐blocker therapy. There is no significant difference in the heart rate at baseline or the change after atropine but significantly longer QT and QT_peak_ adaptation times in those with beta‐blocker therapy. BB, beta‐blocker; T90 End, the time to reach 90% of ΔRR, ΔQT or ΔQT_peak_; τ, the time constant of the exponential function fitted to the reaction in RR, QT, and QT_peak_. *p < 0.05, **p < 0.01.

Although ΔQT and ΔQT_peak_ were 48 and 32% greater in LQT1 patients than controls, respectively (Table 2), the QT adaptation time measures τ and T90 End were on average 25% shorter in LQT1 patients than controls. In contrast, there was no significant difference for the QT_peak_ adaptation times between LQT1 patients and controls (Table 2; Figure 2). Figure 2, however, shows that the partial overlap between patients and controls was due mainly to LQT1 patients on beta‐blocker therapy. Therefore, a post hoc comparison between the 6 LQT1 patients with and the 12 without beta‐blockers was performed. It showed that the baseline HR was slightly but not significantly lower in those on beta‐blockers. The QT and QT_peak_ adaptation times were, however, significantly longer in the beta‐blocker group (Figure 3). In addition, a post hoc analysis excluding the 6 patients treated with beta‐blockers resulted in a reduction of both time measures for QT_peak_ among LQT1 patients (*n* = 12), from 47 (14) to 42 (8) s for τ and from 105 (23 ) to 95 (16) s for T90 End. As a consequence, significant differences between LQT1 patients and controls were observed for both time measures (*p* < 0.05), similar to the results of the QT analysis for the entire group. Exclusion of these six patients resulted in minimal changes of ΔQT and ΔQT_peak_ of 3 ms for each (a 3–4% reduction) without changing the significant differences between LQT1 patients and controls.

## 4 DISCUSSION

The current study tested if and how the hysteresis in QT adaptation to rapid HR increase induced by an atropine bolus injection was affected by loss‐of‐function mutations in the slow component of the outward directed potassium current (I_Ks_) in LQT1 patients in comparison with healthy controls. Although the HR reaction was similar and the mean ΔQT and ΔQT_peak_ were greater in LQT1 patients than in healthy controls, the QT (but not QT_peak_, possibly a beta‐blocker effect; see below) adaptation time was shorter in LQT1 patients. Our results thus indicate that LQT1 is associated with a disturbance in the hysteresis in QT adaptation to increased HR and hence with a dysfunction of the ultra‐rapid cardiac memory (Berger, 2004; Rosen & Bergfeldt, 2015).

Our results showed that a pathogenic loss‐of‐function mutation affecting I_Ks_ was associated with reduced QT adaptation hysteresis (i.e. shorter adaptation time) in LQT1 patients on the group level, although we observed some overlap with healthy controls, especially in patients on beta‐blockers. The QT hysteresis is the result of adaptation in several ion channels for membranous in‐ and outward directed currents (I_Na_, I_CaL_, I_Kr_, I_Ks_) and ion pumps (i.e. Na/K‐ATPase) (Eisner et al., 2009; Pueyo et al., 2010). Because of this complex interaction, of which not all details are completely known, it was far from clear at the start of the study if loss‐of‐function mutations affecting I_Ks_ would alter the QT adaptation hysteresis in humans because of the presence of the repolarization reserve exerted by other potassium currents (Roden, 2006). Our results show that such mutations indeed affected QT hysteresis which corroborates the importance of I_Ks_ in this regulatory mechanism, it seems to be a major functional player. Our LQT1 patients had different mutations in the *KCNQ1*gene with presumably different functional results, which might affect the cellular interactions. This study, however, provides no specific information as to the interaction between the different ion currents and pumps as discussed by Eisner et al. 2009 and Pueyo et al. 2010, an issue that warrants further study.

### 4.1 QT adaptation hysteresis

The hysteresis of QT adaptation to HR change has been studied in several reports by investigating the QT/RR relationship during exercise testing or from Holter recordings, applying different measures of hysteresis with conflicting results as reviewed recently (Gravel et al., 2018). Differences between studies may at least in part be due to the well‐known difference in hysteresis when increasing versus decreasing the HR, faster in the former as shown also in humans (Seethala et al., 2011; Axelsson et al., 2021). This essential feature of QT hysteresis was pointed out (together with other limitations) in one previous very thorough QT/RR study (Malik et al., 2008). For this reason, pacing‐induced HR changes should be preferred over Holter recordings and exercise testing for investigations of QT hysteresis.

Although atrial and ventricular pacing result in similar dynamics in QT hysteresis, ventricular pacing induces potentially confounding repolarization changes related to ventricular activation‐induced short‐term cardiac memory, at least when ventricular pacing lasts 8 minutes or more (Axelsson et al., 2021; Rosen & Bergfeldt, 2015). Incremental atrial pacing is presumably most physiological and was applied in one of our previous studies on patients with supraventricular tachycardia but otherwise healthy undergoing heart catheterization for ablation therapy (Axelsson et al., 2020). Difficulties with keeping 1:1 atrio‐ventricular conduction at a fixed atrio‐ventricular conduction interval and HR turned out to be a problem in that study. Sudden start/stop of atrial pacing was used by Seethala et al. 2011 and in one of our previous studies (Axelsson, Gransberg, Lundahl, Vahedi, & Bergfeldt, 2021). Provided the maximum HR is not too high (in those studies 120 beats per minute), neither keeping 1:1 conduction nor tolerability was a problem. Furthermore, this methodology could be repeated completely non‐invasively in patients with permanent pacing due mainly to sick sinus disease and normal atrio‐ventricular conduction (Axelsson, Gransberg, Lundahl, Vahedi, & Bergfeldt, 2021). However, for reasons stated in the introduction, pacing was not an option in this study.

In attempt to study QT adaptation hysteresis in a standardized way and completely non‐invasively in persons without pacemaker, we used atropine. The doses were high from a clinical point of view, but previously shown to be safe (below age 60) in the clinical setting (Bergfeldt et al., 1996), and in healthy controls (Vahedi et al., 2012), and now also in asymptomatic LQT1 patients. The HR reaction after atropine was similar in LQT1 patients and controls, and we previously showed in the healthy controls that HR after atropine alone was significantly higher than when the beta‐blocker propranolol was administered shortly after atropine (Vahedi et al., 2012). Thus, atropine not only inhibits the parasympathetic influence but also increases sympathetic activity at rest, in accordance with a simultaneous influence of both limbs of the autonomic nervous system on the sinus node at rest shown experimentally already 1934 (Rosenblueth & Simeone, 1934).

Because of the relative lack of parasympathetic innervation of the ventricles, the QT adaptation response was in this study mainly, albeit not entirely, due to the increased HR. In one of our previous studies, isoprenaline was used to increase HR in healthy young people (Vahedi et al., 2012). The sensitivity to isoprenaline, however, varies individually. In that study individualized stepwise increases in the dosage was therefore applied. Consequently, it took much longer time to reach a HR level similar to that after atropine. The time factor and the abundance of beta‐adrenergic receptors in the ventricular myocytes make it likely that the QT adaptation response after catecholamine administration would differ to that after atropine, and the mechanism would be more complex.

Our data on the group level show that the QT but not QT_peak_ adaptation was significantly faster in LQT1 patients than in healthy controls following an atropine‐induced HR increase. The QT_peak_ interval presumably reflects the time for the cells with the earliest complete repolarization in the ventricles of the heart and has a different dynamic than the QT interval, as shown in a recent study on the adaptation following pacing‐induced increase in HR (Axelsson, Gransberg, Lundahl, & Bergfeldt, 2021). When the six patients treated with beta‐blockers were excluded in a post hoc analysis, the QT_peak_ difference in the two time measures between LQT1 patients and controls increased, and became significant, without significantly altering ΔQT_peak_. If beta‐blocker therapy has a more pronounced effect on ventricular myocytes with early (QT_peak_) versus late completion of repolarization (QT) remains an open question. We have no data on QT and QT_peak_ adaptation on/off beta‐blocker therapy.

### 4.2 Clinical/translational implications

The benefit of hysteresis in repolarization adaptation is presumably to provide electrical stability through smooth changes in regional action potential duration (Berger, 2004; Eisner et al., 2009). Further studies are needed to find out if altered QT hysteresis is part of the pathophysiology and arrhythmogenesis in LQT1. In this context, the electro‐mechanical coupling should possibly be considered, since the QT interval roughly corresponds to mechanical systole. Increased HR reduces the diastolic intervals and the time for ventricular filling, which is closely linked to the mechanical output (Franz et al., 1983). In LQT1 patients, symptoms predominantly occur in situations with increased HR (Schwartz et al., 2001), and a too short adaptation time could cause disturbances not only in the electrical function of the heart. The relation between the time for ventricular filling and emptying as well as the time for coronary perfusion would presumably also be negatively affected. Against this background, a combination of disturbed electro‐mechanical function and a mismatch of oxygen demand and delivery might contribute to the propensity for arrhythmias in LQT1 patients in situations with increased HR.

Further studies are also required to determine whether inter‐individual differences in the QT adaptation time in LQT1 patients reflect differences in risk for clinical events, preferably by also studying patients who already have had LQT1‐related events. In light of the increasing number of identified asymptomatic LQT1 patients through family screening, enhanced individualized risk stratification is very much needed, especially since most of them have heart rate‐corrected QT (QTc) intervals less than 500 ms, a threshold value indicating need for therapy. Both disease penetrance and expressivity vary, not only between those with different mutations in *KCNQ1*, but also within a family with the same genetic variant. Adding to the complexity, non‐genetic and genetic factors such as modifier genes influence disease severity and the risk for severe cardiac events (Amin et al., 2013; Schwartz et al., 2012). Risk assessment and therapeutic decisions therefore remain a challenge. Phenotypic characterization of the I_Ks_ function in LQT1 patients, beyond measuring the QTc interval from routine ECG, would therefore be of potential clinical benefit. The arguments for why a “precision medicine” approach with risk assessment based on genotypic and mutation characteristics does not seem to be a solution in LQT1 patients are outlined in the Appendix S1.

### 4.3 Methodological aspects and limitations

The LQT1 patients and the control group were not matched, although the proportions of women and men were similar. There were significant differences in age and blood pressure. The impact of age on repolarization adaptation is unknown. There is, however, experimental evidence that ischemia increases hysteresis (Lauer et al., 2006; Starobin et al., 2007). Therefore, it seems unlikely that higher age and blood pressure in the LQT1 group would lead to shortened QT adaptation time compared with younger healthy controls; the opposite would be more likely. The atropine test does not allow evaluation of the QT adaptation at HR decrease, which recently was found to be slower than at HR increase (Axelsson et al., 2021). For technical reasons, not all recordings could be used for all analyses. Finally, the post hoc observation that LQT1 patients with vs without beta‐blocker therapy had closer to normal QT and QT_peak_ adaptation is primarily hypothesis generating. We do not know if this was due to the therapy itself, but if that shows to be the case, the atropine test might become a test of therapy efficacy.

## 5 CONCLUSIONS

QT adaptation to changes in HR includes a salient feature of hysteresis, a ubiquitous regulatory phenomenon that facilitates a gradual, smooth, and stable adaptation, in this case of electro‐mechanical cardiac function and time for coronary perfusion. The reduction of the QT adaptation time to atropine‐induced HR increase in LQT1 patients on the group level corroborates the importance of I_Ks_ in QT adaptation hysteresis in humans, and on the individual level possibly reflects the effect‐size of the loss‐of‐function mutation, but the clinical implications of these observations need to be shown.

## AUTHOR CONTRIBUTIONS

LB, PD, and K‐JA designed the study; PD, SMJ, FV, LB conducted experiments and acquired data; K‐JA, GL, LG, and LB analyzed data; K‐JA, PD, GL, RP, LG, and LB wrote the manuscript; PD, K‐JA, SMJ, GL, FV, RP, LG, and LB approved the final manuscript.

## FUNDING INFORMATION

This work was supported by the Swedish Heart and Lung Foundation to LB (20190652) and by grants from the Swedish state under the agreement between the Swedish government and the county councils, the ALF‐agreement to LB (ALFGBG‐722431). The sponsors had no role in the study design, data collection and analysis, decision to publish, or preparation of the manuscript.

## CONFLICT OF INTEREST

The authors have no conflicts to disclose.

## Supporting information


Appendix S1
