# Supplementary material for: Accelerated QT adaptation following atropine‐induced heart rate increase in LQT1 patients versus healthy controls: A sign of disturbed hysteresis
Source: Physiol Rep. 2022 Nov 2;10(21):e15487. doi: 10.14814/phy2.15487 (PMC9630760; doi:10.14814/phy2.15487)
Supplement: Supplementary file 1 — Appendix S1 [file PHY2-10-e15487-s001.pdf]

Supplemental Material Dahlberg et al.

Supplemental Figure 1, page 2

Supplemental Figure 2, pages 3-4

Supplemental Table 1, page 5

Supplemental Text, page 6

References, page 7

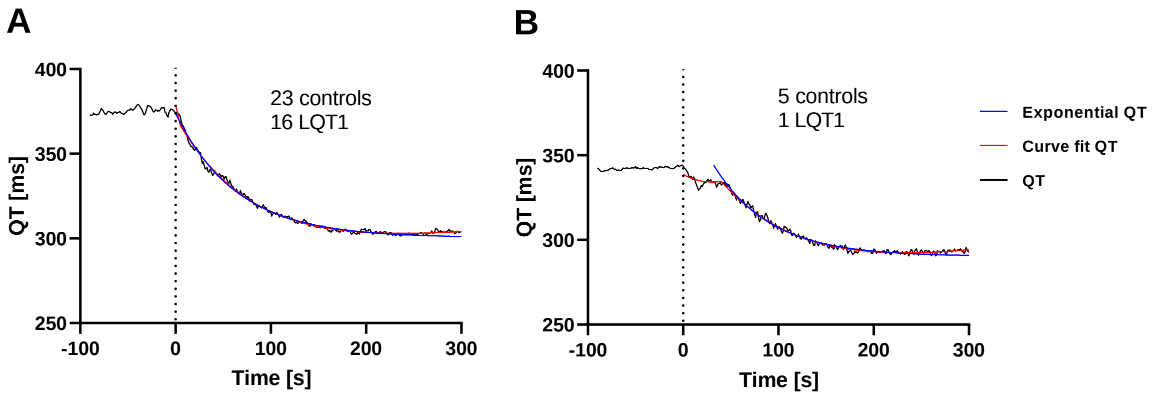

Supplemental **Figure 1**. QT adaptation patterns. In 39 study subjects the mono-exponential adaptation started immediately at time-point 0 (A), while in 6 (1 LQT1, 5 controls), QT adaptation started with a slow phase that could not be fitted to a mono-exponential curve (B). However, after the slow initial phase, the adaptation was mono-exponential in all subjects. Exponential QT: mono-exponential curve fit. Curve fit QT: double exponential curve fit with additional linear components.

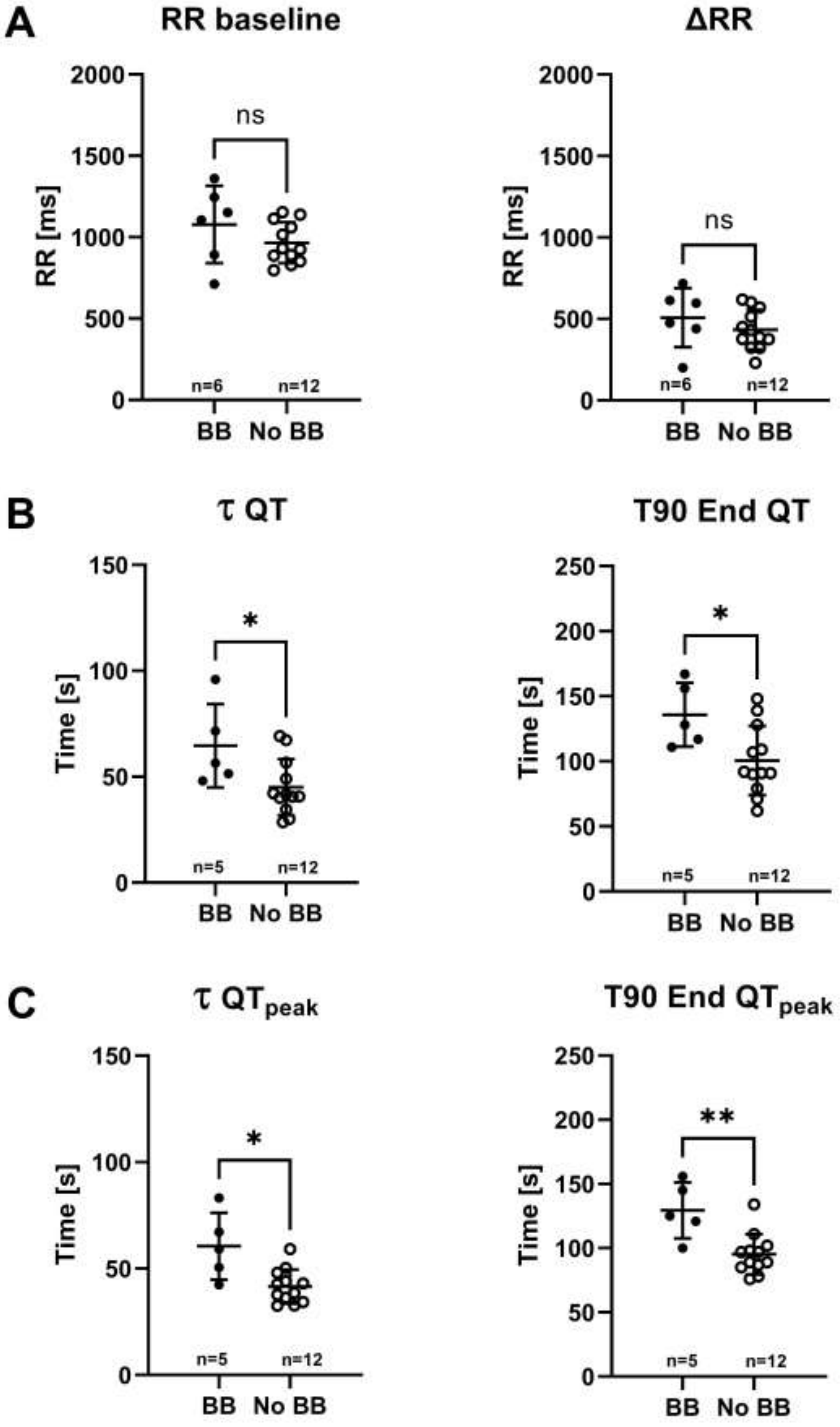

A
RR baseline
RR [ms]
ns
n=6
n=12
BB
No BB
ΔRR
RR [ms]
ns
n=6
n=12
BB
No BB
B
τ QT
Time [s]
*
n=5
n=12
BB
No BB
T90 End QT
Time [s]
*
n=5
n=12
BB
No BB
C
τ QTpeak
Time [s]
*
n=5
n=12
BB
No BB
T90 End QTpeak
Time [s]
**
n=5
n=12
BB
No BB

Supplemental Figure 2. LQTS patients type 1 with (filled circles) versus without (open circles) beta blocker therapy. Comparison of RR (A), QT (B) and QT_{peak} (C) adaptation after atropine injection between LQT1 patients with vs without beta blocker therapy. There is no significant difference in the heart rate at baseline or the change after atropine but significantly longer QT and QT peak adaptation times in those with beta blocker therapy. BB: beta blocker. τ: the time constant of the exponential function fitted to the reaction in RR, QT and QT_{peak}. T90 End: the time to reach 90% of the change in RR, QT and QT_{peak}. $*p < .05$, $**p < .01$

Supplemental Table 1. Atropine-induced changes in instantaneous heart rate (RR interval) and adaptation of ventricular repolarization duration measured as the QT and QT_{peak} intervals in patients and controls with uniform response patterns.

	LQT1		Controls		P-value
	n		n		
**RR baseline [ms]**	17	997 (176)	23	917 (137)	.211
**RR end value [ms]**	17	548 (54)	23	534 (49)	.481
**ΔRR [ms]**	17	449 (142)	23	383 (112)	.141
**τ RR [s]**	17	10 (3)	23	9 (3)	.371
**T90 End RR [s]**	17	23 (7)	23	22 (8)	.343
**QT baseline [ms]**	16	440 (51)	23	378 (29)	<.001
**QT end value [ms]**	16	348 (34)	23	316 (18)	.003
**ΔQT [ms]**	16	92 (24)	23	62 (19)	<.001
**τ QT [s]**	16	51 (18)	23	69 (18)	.003
**T90 End QT [s]**	16	111 (31)	23	148 (30)	.001
**QT_{peak} baseline [ms]**	16	358 (40)	20	303 (27)	<.001
**QT_{peak} end value [ms]**	16	280 (29)	20	245 (15)	.000
**ΔQT_{peak} [ms]**	16	78 (16)	20	58 (18)	.002
**τ QT_{peak} [s]**	16	48 (13)	20	49 (14)	.498
**T90 End QT_{peak} [s]**	16	106 (24)	20	112 (29)	.474

Data shown are the same as in Table 2 but after excluding one patient and 5 controls with different initial response curves as shown in Supplemental Figure 1. Data are shown as mean (SD). Mann-Whitney U test was used to test differences. There were 17 LQT1 patients but one missing for QT and another for QT_{peak}; hence n=16, and 3 controls missing for QT_{peak}; hence n=20. τ: the time constant for the mono-exponential curve fit. T90 End: the time to 90% of the end value of the reaction. bpm: beats per minute

Supplemental text

Discussion

Is precision medicine with risk assessment based on genotypic and mutation characteristics a solution to individualize risk assessment in LQT1-patients?

Applying the falsification principle, the following observations argue against such a possibility. Extensive analyses of an LQT1 founder mutation (A314V) from South Africa showed that the high clinical severity of this mutation could not be fully explained by its location or functional consequence (dominant-negative effect) (1). Similarly, an extensive non-invasive electrophysiological comparison using Frank vectorcardiography at rest of two common Swedish LQT1 mutations Y111C and R518X did not show any major differences, despite their different locations and distinctive differences observed by in vitro electrophysiological evaluation (2). Furthermore, there is a discrepancy between the rather severe reduction in ion channel function at in vitro testing and the relatively low risk for clinical events in LQT1 patients with the Y111C mutation (3). Thus, neither the location of the mutation nor the in vitro functional testing of the I_{Ks} function seems to provide reliable information about the clinical severity in the mentioned LQT1 mutation variants. Furthermore, there are numerous mutation variants in LQT1 which makes it impossible to correlate genotypic and mutation characteristics to the long-term risk for adverse clinical events (4). An attempt to standardize stress-testing of the in situ function of I_{Ks} in the presence of genetic and non-genetic factors whether known or unknown therefore seemed logical. Our study shows that such a procedure is safe and feasible and that it can be performed entirely non-invasively on an outpatient basis, but further studies regarding clinical implications are needed.

References

1. **Brink PA, and Schwartz PJ**. Of founder populations, long QT syndrome, and destiny. *Heart Rhythm* 6: S25-33, 2009.
2. **Diamant UB, Vahedi F, Winbo A, Rydberg A, Stattin EL, Jensen SM, and Bergfeldt L**. Electrophysiological phenotype in the LQTS mutations Y111C and R518X in the KCNQ1 gene. *J Appl Physiol (1985)* 115: 1423-1432, 2013.
3. **Winbo A, Diamant UB, Stattin EL, Jensen SM, and Rydberg A**. Low incidence of sudden cardiac death in a Swedish Y111C type 1 long-QT syndrome population. *Circ Cardiovasc Genet* 2: 558-564, 2009.
4. **Kapplinger JD, Tester DJ, Salisbury BA, Carr JL, Harris-Kerr C, Pollevick GD, Wilde AA, and Ackerman MJ**. Spectrum and prevalence of mutations from the first 2,500 consecutive unrelated patients referred for the FAMILION long QT syndrome genetic test. *Heart Rhythm* 6: 1297-1303, 2009.
